# Metastatic EMT Phenotype Is Governed by MicroRNA-200-Mediated Competing Endogenous RNA Networks

**DOI:** 10.3390/cells11010073

**Published:** 2021-12-28

**Authors:** Sara Uhan, Nina Hauptman

**Affiliations:** Institute of Pathology, Faculty of Medicine, University of Ljubljana, Korytkova 2, SI-1000 Ljubljana, Slovenia; sara.uhan@gmail.com

**Keywords:** EMT, microRNA-200 family, ceRNA, lncRNA

## Abstract

Epithelial–mesenchymal transition (EMT) is a fundamental physiologically relevant process that occurs during morphogenesis and organ development. In a pathological setting, the transition from epithelial toward mesenchymal cell phenotype is hijacked by cancer cells, allowing uncontrolled metastatic dissemination. The competing endogenous RNA (ceRNA) hypothesis proposes a competitive environment resembling a large-scale regulatory network of gene expression circuits where alterations in the expression of both protein-coding and non-coding genes can make relevant contributions to EMT progression in cancer. The complex regulatory diversity is exerted through an array of diverse epigenetic factors, reaching beyond the transcriptional control that was previously thought to single-handedly govern metastatic dissemination. The present review aims to unravel the competitive relationships between naturally occurring ceRNA transcripts for the shared pool of the miRNA-200 family, which play a pivotal role in EMT related to cancer dissemination. Upon acquiring more knowledge and clinical evidence on non-genetic factors affecting neoplasia, modulation of the expression levels of diverse ceRNAs may allow for the development of novel prognostic/diagnostic markers and reveal potential targets for the disruption of cancer-related EMT.

## 1. Introduction

Primary tumor development is a histopathological progression involving the well-documented acquisition of a series of somatic mutations [1,2]. Although relevant, hoping to understand cancer dissemination simply by acknowledging somatic mutations accumulating in the genome of neoplastic cells and defining the genes and gene products that drive the process bears limitations. While mutations in driver genes do facilitate the development of pro-metastatic traits, the baffling complexity of the mechanism seems to originate from crosstalk between regulatory networks. The recent revelation that less than 2% of the human genome is related to protein-coding genes has thus emphasized the importance of unravelling these networks beyond frontline transcriptional control in order to fully comprehend cancer dissemination [3]. Here, we set out to examine the dynamics of the main intrinsic regulatory mechanisms governing cancer-related epithelial–mesenchymal transition (EMT) and identify the stoichiometry of vital players in the competing endogenous (ceRNA) network with a focus on cancer progression and metastasis.

## 2. Epithelial–Mesenchymal Transition

**Figure 1:**
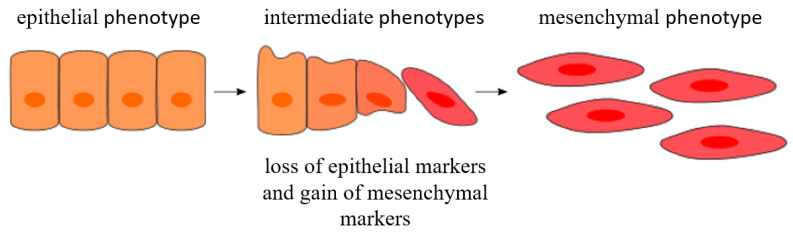
The process of epithelial–mesenchymal transition. A layer of polarized epithelial cells transitions toward a mesenchymal state. The cells with intermediate phenotype have transitioned into a partial EMT state and express both epithelial and mesenchymal markers (adapted from [10]).

The EMT process is a vital cell mechanism which occurs during normal embryonic development, wound healing, and tissue regeneration [4]. Upon EMT, cells shed pre-existing epithelial surface-barrier and secretory functionalities and acquire a series of mesenchymal characteristics (Figure 1) [5]. Theoretically, at the extreme epithelial axis pole (E), epithelial cells exhibit cell-to-cell junctions and polarity, while cells at the extreme mesenchymal pole (M) exhibit heightened invasiveness and motility and spindle-like morphology [6]. Transition occurs through distinct molecular processes (thoroughly reviewed elsewhere [7,8,9]), inclusive of the induction of EMT transcription factors (EMT-TFs), changes in the cell surface protein expression profile, re-organization of the cytoskeleton, and altered expression of specific cellular mediators [10]. In addition, regulation of the EMT process is exhibited through three finely tuned epigenetic molecular layers: (1) small non-coding RNAs, (2) differential splicing, and (3) translational/post-translational control [11]. Importantly, carcinoma cells co-opt these mechanisms and the tumor microenvironment to promote metastatic expansion [12].

Carcinomas, malignancies of epithelial cells, amount to approximately 90% of all human cancers and account for the majority of metastasis-related deaths [13]. Although the complete molecular and morphogenic regulatory mechanism underlying the metastatic potential of carcinoma cells remains elusive, the process of metastatic progression has been well characterized. Upon dissemination, primary neoplastic cells escape tumor borders upon EMT activation, invade the surrounding extracellular matrix, travel through the systemic circulation, extravasate in the parenchyma of a distant organ, and establish secondary tumors [14]. Through this transition, a radical change in function, differentiation, and cell lineage occurs [15].

This biochemical and morphological transition toward a variety of pathologic and non-pathologic conditions presumably operates on multiple regulatory layers [15,16]. Despite increasing efforts to unravel the specifics of the EMT process in neoplastic cells, research into genes with recurrent mutations in metastasized cells is ongoing [8]. Thus, the distinct capabilities of cells acquired throughout the EMT process seem to be independent of concomitant alterations in DNA, but rather point to a coordinated collaboration of epigenetic signaling components [9,17]. A multitude of transcriptional repressors derived from tumor-associated reactive stroma play crucial roles in the regulation of signaling pathways affecting neoplastic cells and inducing EMT, including the zinc finger E-box binding homeobox 1 (ZEB) protein family, the Twist protein family, and the Snail protein family [9,16,18,19,20,21,22,23,24,25]. Specifically, increased expression of EMT-TFs leads to transcriptional repression of CDH1, encoding for junction protein E-cadherin with a central role in the maintenance of the polarized epithelial monolayer and metastatic suppression during tumor progression [16,26]. While the knowledge of TFs acting in EMT reveals many signaling pathways for the induction of phenotypic change in pathogenesis, endogenous expression of EMT-TFs has been found in multiple tissue types [11]. Thus, coupling EMT-TFs exclusively to pathogenic de-differentiation might not always be accurate as these TFs bear broader physiologically relevant roles. With this in mind, compelling data suggests that a disturbance in the balance between different regulatory networks resulting in a shift from epithelial to mesenchymal cell phenotype is one of the essential prerequisites allowing for the invasion–metastasis cascade, differentiating it from the physiologically relevant EMT process.

## 3. Epithelial–Mesenchymal Transition Phenotype In Vivo

Depending on the signaling profile, cells progress through the EMT spectrum of changes in a diverse manner and advance to variable extent toward the mesenchymal phenotype (Figure 1) [8,27]. Interestingly, it has been recently indicated that stable cell residence at any of the extreme poles—either the epithelial (E) or mesenchymal (M) pole—prevents the acquisition of stemness, one of the central qualities a cell acquires in the transition to a mesenchymal phenotype [28]. Moreover, the optimal state for acquiring stem cell capabilities was shown to be the intermediate E/M cell state, termed partial EMT [29]. Kröger and colleagues showed that forcing highly tumorigenic E/M hybrid cells to undergo full transition toward the extreme M state, a process referred to as complete EMT, results in a loss of tumorigenicity [30]. Furthermore, Tsai and colleagues [31] demonstrated that the transient nature of EMT promotes tumor cell dissemination in vivo. A similar conclusion was drawn for circulating tumor cells with an intermediate E/M phenotype, as they could exit the bloodstream more efficiently than cells with the extreme E or M phenotype, and consequently pose a higher metastatic risk [32]. The plasticity of the process indicates that EMT-related carcinoma progression does not function as a binary switch between the two extremes. Rather, carcinoma cells rarely (if ever) execute the complete EMT program in a spontaneously arising tumor [8,33]. Even though complete EMT can be achieved experimentally, this is likely to be a non-physiological phenomenon arising mostly as an experimental artefact, which was demonstrated in multiple in vivo studies [34,35,36]. In spindle cell carcinoma, varying patterns of cadherin expression indicate that EMT indeed comprises a wide spectrum of changes, where complete transition results in a loss of epithelial and gain of mesenchymal markers [15]. Furthermore, during partial EMT, some cells fail to activate the mesenchymal gene expression program [37]. As EMT-related molecular and phenotypic changes are transient and non-conclusive for all tumor cells, this might be the reason behind the incapacity to prove complete EMT on a molecular level in vitro. In conclusion, the fluidity of EMT is evident in the co-expression of both epithelial and mesenchymal markers on intermediate hybrid E/M cells, where partial EMT might represent the final cell differentiation state.

## 4. Competing Endogenous RNA Hypothesis

**Figure 2:**
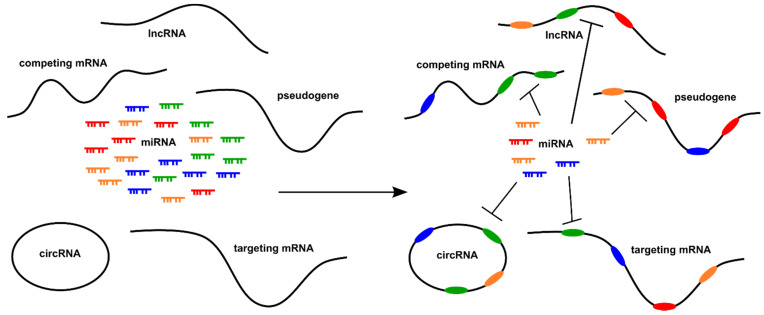
Network of competing endogenous RNAs (ceRNAs). MicroRNAs bind to miRNA response elements (MREs) that are in the transcriptome of different classes of RNA transcripts, namely circRNAs, lncRNAs, pseudogenes, and protein-coding mRNA. All classes of RNA transcripts contain MREs and compete for miRNA binding, thereby regulating the common pool of miRNA. All represented classes carrying the same MRE interact with each other through competition for the same miRNA (adapted from [40]).

Competitive regulatory crosstalk of different molecular species, including DNA–protein, RNA–protein, RNA–RNA, and protein–protein interactions, has been described in many non-pathological as well as pathological settings [38,39]. This review focuses on sequence-based competition of naturally occurring protein-coding and non-coding RNA transcripts, termed here as competing endogenous (ceRNAs) RNA molecules, for microRNA (miRNA) molecules, resulting in aberrant gene expression regulation (Figure 2).

MiRNAs are an abundant class of small, non-coding, single-stranded oligoribonucleotides, serving as sequence-specific silencers of target gene transcription upon binding [41]. They serve as regulators in many diverse cellular processes and their expression profile ranges from being largely ubiquitous to highly temporal or site-specific [26,38]. It was recently estimated that miRNAs affect expression levels of more than half of all protein-coding genes [42]. However, functional studies often show only a modest decrease in target gene expression [43]. A reason for this can be found in the ubiquitous character of miRNA molecules for all biological networks. The majority of RNA molecules act in a “many-to-many” manner—each miRNA molecule can potentially target miRNA response elements (MREs) on multiple messenger RNA (mRNA) molecules, while each mRNA molecule can be targeted by multiple miRNA molecules [44]. This principle explains the competition for small regulatory molecules among multiple target transcripts and designates miRNA molecules as having a central role in the competitive regulatory crosstalk.

The ceRNA hypothesis suggests that the competition for miRNAs encourages functional interactions among common targets to induce post-transcriptional regulation [45,46,47]. As postulated, ceRNA acts in a competitive manner [45,47]. When the expression levels of a specific ceRNA increase, the probability of it binding to the target miRNA increases as well, leading to a decrease in miRNA expression levels, which results in decreased miRNA–mRNA binding. Consequently, an increase in mRNA expression levels can be detected. Similarly, reverse effects can be seen with low ceRNA expression levels [45,47]. Arvey and colleagues [48] proposed that miRNA-guided post-transcriptional regulation does not solely depend on their binding sites and the quantity of target molecules [48]. They found that many RNA molecules are able to titrate specific miRNAs from the common pool, consequently sequestering them away and causing upregulation of the target gene. This competitive race amongst diverse regulators with common target sequences leads to the assumption of functional redundancy of some RNA families.

## 5. Competing Endogenous RNA Network

CeRNAs competing for common MREs regulate one another via ceRNA networks (ceRNETs) [49,50,51]. CeRNETs generally consist of two different levels of regulation: (1) direct competition between two ceRNA regulators sharing common MREs, and (2) indirect linkage via a common ceRNA, independent of miRNAs, through which a titration mechanism occurs [51,52,53]. The modeling work undertaken by Ala et al. [53] suggested that proportional balancing of miRNAs and other ceRNAs is crucial in gene expression regulation, in which alterations often lead to pathological states. They demonstrated that an increase in a specific ceRNA can result in a proportional increase in the expression of another regulatory ceRNA, together with a decrease in available miRNA levels. Furthermore, they implied that losing a single ceRNA regulator and changing the near equimolar equilibrium may result in changes on a global level, since multiple two-part ceRNA crosstalks were found to be affected by a third transcript, leading to a critical alternation. Although several regulatory networks have been proposed for different species of RNA that offer a clearer view of their functionality in cancer [54], a genome-wide scale study to define the intertwined roles of non-coding transcripts and potential non-coding functions of coding genes would be critically beneficial.

### 5.1. MicroRNA-200 Family and ZEB Factors

The pivotal role in cancer-related EMT has been continuously assigned to the microRNA-200 (miR-200) family [55,56,57]. The miR-200 family comprises five members that can be further divided into two functional subgroups based on a single-base difference in an otherwise conserved Watson–Crick pairing of the 5′ region of the miRNA: (1) miR-200a and miR-141, further referred to as subgroup I, and (2) miR-200b, miR-200c, and miR-429, further referred to as subgroup II [10,15,41]. The MiR-200 family shows enriched expression in tissues where the epithelial phenotype prevails [58,59], and has been associated with the initiation and progression of malignant transformation [24,60]. Aberrant expression of miR-200 members results in tumor progression by lowering the expression of vital surface and polarity factors [61]. A study on spindle cell carcinoma showed significant miR-141, -200a, -200c, and -429 downregulation in carcinoma tissue compared with normal mucosa [15,62]. Furthermore, downregulation of the miR-200 family showed a significant correlation with loss of E-cadherin, characteristic for EMT progression [15,63,64,65]. Another study demonstrated that restored levels of miR-200a inhibited tumor growth in vivo [66]. Nevertheless, as a consequence of intra-tumoral heterogeneity, the causal relationship of aberrant miRNA expression and its pathophysiological relevance in cancer is difficult to assess.

Specificity of the potent regulation of miRNA species is exerted through a 2-7-nucleotide long “seed sequence” in the 5′ end, binding by complementarity to the MREs in the 3′ UTR of the target mRNA [60]. Common binding targets for the miR-200 family are the conserved miR-200 sites in the 3′ UTR of the repressors of E-cadherin expression, ZEB1 and ZEB2, inducing EMT by limiting the expression of genes crucial for development and maintenance of the epithelial cell phenotype [10,55,67]. Interestingly, all members of the miR-200 family were found to be transcriptional targets of ZEB1 and ZEB2 [68], displaying a reverse inter-relation and proposing a double-negative feedback loop [24]. The reciprocal relationship of the regulatory circuits was described by many, implying a detrimental effect of the opposing functionalities, possibly resulting in miR-200 family downregulation and ZEB1 upregulation, both in support of EMT promotion [69,70,71,72,73].

### 5.2. LncRNA and miRNA Subgroup I

Long non-coding RNA molecules (lncRNAs) were recently found to be involved in the regulation of many biological functions as well as cancer progression [45]. Accounting for the largest portion of the human transcriptome, lncRNAs are involved in diverse biological functions [74]. While miRNAs were found to depend mostly on the abundance of their targets, target multiplicity of lncRNA molecules is ascribed to cross-regulation in the common pool of MREs [48]. In support of this, lncRNA were indeed found to sponge and hijack the miRNA pool that targets transcripts with essential roles in EMT, including E-cadherin [46,74]. While we now know that some lncRNA molecules function as oncogenes and others act as tumor suppressors [54], the functions and mechanisms of the crosstalk between lncRNA, circular RNA (circRNA), pseudogenes, and other types of RNAs remain unclear [45].

Elucidating the diverse roles of lncRNAs is a major focus in cancer research since their role of regulating target mRNA expression through competitive binding to shared miRNA sites has been confirmed [75]. Accumulating data point to lncRNAs having a vital role in sponging miRNAs in EMT and consequently sequestering away specific molecules and their functionality. The tilting of the balance toward one of the EMT poles is activated through the regulation of surface protein expression, EMT-TFs, and players in the ceRNA crosstalk. There are a few common characteristics of every lncRNA molecule in a specific ceRNET [47]. Firstly, lncRNAs were found to be the primary targets of miRNA; they show a competitive character toward miRNAs for common mRNAs in ceRNETs [74]. Furthermore, the ceRNA hypothesis provides a justification for both the functionality of lncRNAs in the network as well as an explanation for the 3′ UTR regulatory function [47].

H19 was the first molecule to be identified as lncRNAs [76]; since then, its role in various cancers has been established [24,55,58,71,77,78,79] (Table 1). Furthermore, H19 was shown to be essential for tumor metastasis [80,81] and was found to be involved throughout the process of tumorigenesis [82]. H19 facilitates the invasion of colon cancer cells via miR-200a sponging mechanism and consequential ZEB1/2 upregulation, while its overexpression results in upregulation of vimentin and downregulation of E-cadherin, and indicates a significant increase in tumor size in vivo [83].

While extensive research has been done on the role of H19 lncRNA in cancer metastasis since its discovery, several other lncRNA molecules were recently found to be critical in the regulation of cancer-related EMT (Table 1). Metastasis-associated lung adenocarcinoma transcript 1 (MALAT1) lncRNA molecule is one of the most commonly implicated lncRNA molecules in various types of cancer; Xiao et al. further defined its role as an oncogene, acting through sponging of multiple miR-200 family members, resulting in ZEB2 increase. They demonstrated that a knockdown of MALAT1 decreased renal cancer cell proliferation, migration, and invasion potential in vitro and in vivo [86]. A recent study performed by Wei and colleagues showed significant downregulation of miR-200a with an upregulation of MALAT1 lncRNA molecule in non-small cell lung cancer, which indicates that the MALAT1/miR200a/PD-L1 axis might have an important role in EMT [84]. Similar results were found in anaplastic thyroid carcinoma, where disease progression is regulated by MALAT1-dependent sponging of tumor suppressor miR-200a and results in FOXA1 oncogene upregulation [85]. Importantly, programmed death-ligand 1 (PD-L1) was found to arrest metastasis of non-small cell lung cancer cells; its expression is directly affected by miR-200a and regulated by the MALAT1/miR200a/PD-L1 negative feedback loop [84]. As functions of MALAT1 lncRNA differ among cancer types [111,112,113], this might imply that specific final effects may depend on specific molecular interactions [84].

LncRNA TP73 antisense RNA 1T (TP73-AS1) plays a crucial role in many different carcinomas [95]. As miR-200a was found to directly inhibit expression of human mitochondrial transcription factor A (TFAM), coding for a protein involved in breast cancer cell proliferation, reduced TFAM levels were detected in a TP73-AS1 knockdown experiment, where inducing miR-200a inhibition resulted in restitution of primary protein levels [97]. Likewise, aberrant expression of ZEB1 TF in EMT is a consequence of ZEB antisense 1 (ZEB1-AS1)-induced miR-200a sponging, resulting in EMT promotion in intrahepatic cholangiocarcinoma in vitro and in vivo [99]. Similarly, Bacteroides fragilis-associated lncRNA1 (BFAL1), participating in gut bacteria-induced carcinogenesis, was found to bind to miR-200a in a competitive manner, activating the MTORC1 binding/mammalian target of the rapamycin (RHEB/mTOR) pathway, which is commonly dysregulated in cancer [102]. With tumor-suppressing miR-200a as its target in papillary thyroid carcinoma, small nucleolar RNA host gene 15 (SNHG15) indirectly regulates expression of miR-200a’s target YAP1 oncogene and enhances disease progression [88]. Furthermore, small nucleolar RNA host gene 16 (SNHG16) was proposed to regulate the balance in an SNHG16/miR-200a ceRNET and was associated with regulation of malignancy potential of colorectal cancer cells [101]. Deregulation of highly upregulated in liver cancer (HULC) lncRNA molecule in several cancer types has been associated with the PI3K/AKT signaling pathway, which is central to cell proliferation, differentiation, and apoptosis [103]. Upon miR-200a-related HULC silencing in leukemic cells, the number of apoptotic cells dramatically increased, while HULC upregulation resulted in leukemic cell proliferation, apoptosis resistance, and cell cycle progression, all attributed to aberrant PI3K/AKT signaling and an increase in c-Myc and Bcl-2, two critical cell survival regulators [103].

Aberrant expression patterns for H19, MALAT1, TP73, ZEB1-AS1, SNHG15, and others were detected in a multitude of other cancer types [95,114,115], as well as in relation to other molecules belonging to the microRNA-200 family. H19 was found to regulate miR-141 in ceRNA crosstalk and plays an essential role in human breast cancer tumor metastasis [116]. Similarly, ZEB1-AS1 was found to sequester miR-141 in colorectal cancer [100]. SNHG15 expression correlates with aberrantly expressed programmed cell death ligand 1 (PD-L1), where NHG15/miR-141 interaction results in upregulation of PD-L1, allowing gastric cancer cells to escape the host’s immune response [93]. Similar SNHG15-dependent sequestering of miR-141 has been associated with the progression of multiple cancer types, including hepatocellular carcinoma [89], colorectal carcinoma [90], osteosarcoma [91], and nasopharyngeal carcinoma [92]. In addition, Li and colleagues revealed the co-expression of MAGI2 antisense RNA 3 (MAGI2-AS3) with ZEB1 and identified their role in the promotion of gastric cancer through miR-141/200a sponging [87]. Future studies on MAGI1-AS3 transcriptional regulators and expression trajectories will hopefully further understanding of the role of MAGI1-AS3 in the ceRNA cancer network.

### 5.3. LncRNA and miRNA Subgroup II

Through the accumulation of knowledge on ceRNA networks, we are beginning to understand that post-transcriptional manner of action plays an essential role in cancer metastasis regulation and depends on interactions between specific ceRNA molecules. Zhou and colleagues hypothesized that H19 physically associates with miR-200b and miR-200c to construct a ceRNA sponge and indirectly regulates expression levels of Git2 and Cyth3, two regulators of the RAS superfamily member adenosine 5′-diphosphate ribosylation factor (ARF) [80] (Table 2). ARF promotes EMT and cell migration of tumor cells; H19 critically contributes to a change in ARF abundance by sponging its regulators [80]. Oncogenic OIP5-AS1 lncRNA was found to affect fibronectin-1 (FN1), a glycoprotein with a vital role in cellular adhesion in migration processes [117,118]. Through miR-200b sponging, the EMT process is affected by aberrant levels of FN1 expression; in addition, FN1 was found to be significantly upregulated in chemo-resistant osteosarcoma tissues [118]. Wu and colleagues identified the role of OIP5-AS1 in the facilitation of cell growth in vitro as well as in vivo through miR-429/FOXD1 axis regulation [119].

Long non-coding RNA activated by transforming growth factor β (lnc-ATB) was found to be abnormally expressed in a number of cancer types and exerts pro-metastatic traits by competitively binding miR-200c [129] as well as miR-141 [110,134]. This was shown to result in the upregulation of EMT progression-related genes, including ZEB1 [134] and ZNF-217 [129]. In addition, when lnc-ATMB expression was silenced in a knockdown experiment, consequential downregulation of vimentin and N-cadherin and upregulation of E-cadherin were detected [128]. Furthermore, Li and colleagues confirmed the binding of miR-200c to the 3′UTR of the MALAT1, forming a sponge in endometrioid endometrial carcinoma [120].

In hypopharyngeal squamous cell carcinoma tissues, both MALAT1 and ZEB1 were upregulated, while miR-429 levels decreased [122]. Furthermore, evidence of suppressed in vitro cell proliferation, migration, and invasion [122,124], in addition to repressed cell growth in vivo [122,135], indicate a central role of MALAT1 in the promotion of cancer cell migration and disease progression through miR-429 sponging. Wang and colleagues showed the involvement of long intergenic non-protein-coding RNA 667 (LINC00667) in ceRNA by occupying the binding motif of miR-429, which lead to the promotion of cancer cell proliferation, migration, and invasion [136]. Furthermore, this molecule was shown to act as a sponge for miR-143 [137] and miR-200c [138], decreasing their expression and inhibiting their functionality, and has a role in promotion of EMT by suppressing E-cadherin expression. Liu et al. demonstrated the primary involvement of LINC00667 in Wilms’ tumor metastasis by sponging miR-200b/c/429 and modulating critical regulatory and signaling pathways [139]. Similarly, silencing of DLEU1 inhibited migration and invasion of ovarian cancer cells through targeted sponging of miR-429 [133]. Importantly, overexpression of miR-429 restored the inhibition of proliferation, migration, and invasion of ovarian cancer cells [133,140].

### 5.4. CircRNA

CircRNAs are a distinct ceRNA subtype, different from lncRNAs in their closed-loop structure [141]. They are known for their stability and resistance to ribonucleases [141]. While the complete function of most circRNAs is yet to be defined, the functionality of some circRNAs has been unraveled recently. Zhu and colleagues showed how silencing of circ-0067934 limits the proliferation, migration, and invasion of hepatocellular carcinoma, while suppression of miR-1324 through binding induces cell apoptosis [142]. Furthermore, the frequently downregulated miR-615-5p, known to act as a tumor suppressor, is regulated post-transcriptionally by both circRNAs and lncRNAs [143].

While hsa_circ_0008305 was found to inhibit EMT and metastasis in non-small cell lung cancer and offers a potential therapeutic strategy [144], most circRNAs show miRNA sponging functionality and therefore promote EMT (Table 3). A study from 2019 [145] revealed aberrant expression of hsa_circ_0057481 in malignant tissue from patients with laryngeal cancer and was found to affect miR-200a/miR-200c-associated regulation of ZEB1, while aberrantly expressed hsa_circ_001783 downregulated miR-200c expression, thus inducing EMT and resulting in poorer prognoses in breast cancer patients [146]. Knockdown of circ_GNB1 significantly decreased the expression of IGF1R [147], a protein which is aberrantly expressed in malignancies and enhances cell survival, and is thus considered a potential therapeutic target [148,149]. As circ_GNB1 sponges miR-141 and upregulates IGF1R [147], these results demonstrate the detrimental role of this circRNA molecule in this ceRNET. Similarly, Li and colleagues identified an important circRNA–miRNA–mRNA axis which modulates the migration potential of hepatocellular carcinoma cells. Upon circ_101368 knockdown, miR-200a expression was rescued, which decreased the migratory activity of hepatocellular carcinoma cells through HMGBI/RAGE signaling [150].

In conclusion, these results confirm that circRNAs represent important modulators of mRNA expression through miRNA sponging. Promotion of proliferation due to de-regulated levels of miRNA, achieved via circRNA sponging, has been detected for many other circRNA molecules and could serve as a prognosis indicator system in a clinical setting [154].

### 5.5. Pseudogenes and mRNA

Pseudogenes were found to be an additional class of regulatory molecules in the EMT. The term pseudogenes describes DNA sequences that are similar to the functional gene but contain non-transcribed nucleotide sequences that result from terminated translation [47]. Their processed versions form through retro-transcription of spliced mRNA into DNA and its re-integration into the genome [157]. Due to the high sequence homology between pseudogenes and their protein-coding gene counterparts, the overlap represents a common target pool for the same miRNAs [47,158]. In fact, some of the earliest evidence for ceRNA activity in mammalian cells was revealed when analyzing the haploinsufficient tumor suppressor pseudogene 1 (PTENP1) and its parental gene, haploinsufficient tumor suppressor (PTEN) [159]. Since the loss of the PTENP1 locus is accompanied by reduced PTEN expression in melanoma [158] and colon cancer [159], PTENP1 has been shown to regulate PTEN post-transcriptionally and act as a decoy for PTEN-targeting mRNA [159,160]. Additional pseudogene regulators were confirmed to have the ability to act as decoy miRNAs for their gene counterparts [161,162]. Similar to other ceRNA molecules, pseudogene-derived RNA molecules can promote initiation of EMT as cross-regulators by sponging miRNAs [163]. While over 10,000 pseudogene molecules have been identified [164], knowledge on their biological importance is lacking, specifically in relation to the miR-200 family. Further investigation into pseudogene biology and regulation are essential to establish which mRNA, miRNA, and protein molecules crucially affect the induction of EMT, potentially acting as competitive endogenous sponging molecules for pathogenic expression of pseudogenes.

Since the revelation that a single dynamically induced mRNA could regulate EMT by inducing MREs [46], mRNA is considered to be an active participant in the regulatory network rather than a passive target, as commonly perceived. Due to high expression levels of specific mRNAs and lowered levels of miRNA expression in cancer cells, compelling evidence shows how a single aberrantly expressed ceRNA is directly coupled with the double-negative feedback loop in EMT [46]. The ceRNA–mRNA stoichiometry could represent a crucial parameter that determines both the reversibility and the stage of EMT. However, greater knowledge on binding affinity, time, location, and energy—and its appropriate assessment—is needed in order to understand how mRNA levels affect ceRNETs.

## 6. Conclusions

Since the first postulation of the ceRNA hypothesis, the functional roles of ceRNAs in neoplastic progression have been reported in a series of studies [49,158,161]. Molecules acting in ceRNETs have revealed differential expression in tumor compared with in normal samples [47]. CeRNA activity closely resembles a large-scale regulatory network of gene expression controlling EMT and expanding the functional genetic information across the human transcriptome. While miRNAs have been conventionally considered to be mRNA regulators, there is evidence of back-regulation through changes in target mRNA availability due to sponging by ceRNET members. Precisely this broader perspective allows for the hypothesis of a double-negative forward loop, in which the miR-200 family is regarded as a segment in cancer-related EMT, rather than an individual regulator. The stoichiometry between miRNAs and other ceRNA allows dynamic transition between epithelial and mesenchymal phenotypes, which can be achieved by manipulating a single member of the ceRNA network [24].

It is now becoming clear that de-regulation of lncRNA, miRNA, mRNA, pseudogenes, circRNA, and other ceRNA molecules can result in tumorigenesis. Whether dynamic changes in ceRNA stoichiometry can modulate tumor suppression of miRNA activities is still a matter of controversial dispute, mostly focusing on in vitro vs. in vivo molecular abundance discrepancies [165]. Another point of controversy is related to the proportion of transcription and degradation of miRNA and targets—due to binding time and affinity, the abundance of miRNA is the key regulator in ceRNETs [165]. Nevertheless, the ceRNA hypothesis offers an interesting and relevant view on epigenetic regulation, portraying RNA molecules as important modifiers of the epigenetic landscape instead of merely “bearers of genetic information”. Upon acquiring more knowledge and clinical evidence, distinct ceRNA members could serve as prognostic/diagnostic markers as well as potential targets for the disruption of uncontrolled EMT in neoplastic progression [115,126,153].

Furthermore, this work directs attention toward a parallel controversial issue—the spectral, bi-phenotypic EMT status of cells in transition. Conclusions as to whether reports of the existence of a partial EMT cell state in vivo [166] describe a transitional state (i.e., an intermediate stage between the transition from the E to the M pole) rather than a finalized state in the cell differentiation process have not yet been drawn. The complexity of the ceRNA network reflects the multifaceted character of EMT with ceRNA sponging of miRNA directly affecting the extent of transition toward one of the differentiation poles. The ceRNA hypothesis allows for system-level effect analysis, taking into account both feed-forward and feed-back miRNA–mRNA loops and regulation by other molecular species, as well as the possibility of coding-independent regulatory functions of genes.

### Outlook

Dysregulation of the ceRNA network notably plays a critical role in promotion of the invasion–metastasis cascade and can lead to the development of a variety of tumors [74]. Thus, quantitative change in the miRNA population, leading to a decrease in mRNA targeting, bares important implications in defining the causal actors in the crosstalk. Due to an unmet need for early diagnosis and prognosis in various cancers, ceRNA crosstalk remains an important avenue worth exploring. While chemotherapy and immunotherapy remain the standard-of-care treatment, it has been recently demonstrated that inhibition of specific ceRNA components could, in the future, represent an alternative therapeutic target for the treatment of certain cancer types. In addition, ceRNA was reported to be functional in a therapeutic resistance setting [167], rendering it a potential prognostic and diagnostic tool.

While we are still in the early stages of translating the acquired knowledge on ceRNA regulation into the clinical setting, important steps have been made to advance this transition. Importantly, through improved understanding of miRNA biogenesis, regulation, and alterations, alongside simultaneous advances in technologies to deliver miRNA-based therapeutics in vivo, we are now able to divide miRNA-based therapeutics into candidates for therapeutics (miRNA mimics) or targets (antimiRs) [168]. In the case of miRNA mimics, the aim is to replenish the pathologically induced decrease in miRNA expression by designing double-stranded RNA molecules matching the target mRNA. In contrast, antimiRs are single-stranded molecules which are complementary to the dysregulated miRNA sequence and therefore prevent its functionality [168]. In addition to the complexity of identification of miRNA target candidates, one of the main challenges on the path toward clinical application are the limitations in delivery system options. Due to the nature of RNA molecules, RNA-based therapeutics face the potential of undesired degradation by RNases in the serum or endocytic compartment of cells [168]. Additionally, with an ideal delivery system, off-target toxicities should be avoided. Genome-wide functional screens using miRNA mimics or inhibitors, identification of miRNAs that are able to re-sensitize chemoresistant cancer cells, and extensive research on the miRNA “targetome” related to specific oncogenes and tumor suppressor genes are just a few of the avenues in need of attention for advancement toward the use or miRNA-based therapeutics. With recent advancements in genomics, sequencing approaches, and capture techniques for more precise miRNA “targetome” identification, a more profound understanding of the ceRNA concept could help to translate these findings into applications in the clinical setting.

## Figures and Tables

**Table 1:** Members of lncRNA-miRNA-mRNA ceRNA network type for miRNAs in subgroup I.

ceRNA Member	Competitor	Shared miRNA	Model Type	Cancer Type	Ref.
MALAT1	PDL1	miR-200a	in situ, in vitro	non-small cell lung cancer	[84]
	FOXA1	miR-200a	in vivo, in vitro	anaplastic thyroid carcinoma	[85]
	ZEB2	miR-200 family	in situ	clear cell kidney carcinoma	[86]
MAGI2-AS3	ZEB1/2	miR-200a/miR-141	in situ	gastric cancer	[87]
SNHG15	YAP1	miR-200a	in situ, in vitro	papillary thyroid carcinoma	[88]
	ZEB2/E2F3	miR-141	in situ, in vitro	hepatocellular carcinoma	[89]
	SIRT1	miR-141	in situ, in vitro	colorectal cancer	[90]
	/	miR-141	in situ, in vitro	osteosarcoma	[91]
	KLF9	miR-141	in vitro	nasopharyngeal carcinoma	[92]
	PDL1	miR-141	in situ, in vitro	gastric cancer	[93]
H19	ZEB1/2	miR-200a/141	in situ, in vitro	lung cancer	[94]
	VIM/ZEB1/ZEB2	miR-200a	in situ, in vitro	colorectal cancer	[83]
TP73-AS1	BDH2	miR-141	in situ, in vitro	pancreatic cancer	[95]
	HMGB1/RAGE	miR-200a	in situ, in vitro	hepatocellular carcinoma	[96]
	TFAM	miR-200a	in situ, in vitro	breast cancer	[97]
	ZEB1	miR-200a	in situ, in vitro	breast cancer	[98]
ZEB1-AS1	ZEB1	miR-200a	in situ, in vitro	intrahepatic cholangiocarcinoma	[99]
	/	miR-141	in situ, in vitro	colorectal cancer	[100]
SNHG16	/	miR-200a	in situ, in vitro	colorectal cancer	[101]
BFAL1	RHEB/mTOR pathway	miR-200a	in situ	colorectal cancer	[102]
HULC	c-Myc/Bcl-2 (PI3K/AKT pathway)	miR-200a	in vitro	chronic myeloid leukemia	[103]
MRAK081523	Plxna4	miR-141	in vivo, in vitro	pulmonary fibrosis	[104]
HOTAIR	SKA2	miR-141	in situ, in vitro	glioma	[105]
linc00475	YAP1	miR-141		glioma	[106]
LINC01857	MAP4K4	miR-141	in situ, in vitro	diffuse large b-cell lymphoma	[107]
MIAT	SIX1/PI3K/AKT pathway	miR-141	in situ, in vitro	osteosarcoma	[108]
	DDX5	miR-141	in situ, in vitro	gastric cancer	[109]
XIST	/	miR-141	in situ, in vitro	colorectal cancer	[110]

**Table 2:** Members of lncRNA-miRNA-mRNA ceRNA network type for miRNAs in subgroup II.

ceRNA Member	Competitor	Shared miRNA	Model Type	Cancer Type	Ref.
MALAT1	MET markers	miR-200c	in vivo	endometrioid endometrial carcinoma	[120]
	/	miR-200c	in situ, in vitro	ovarian cancer	[121]
	ZEB2	miR-200 family	in situ	clear cell kidney carcinoma	[86]
	ZEB1	miR-429	in situ, in vitro	hypopharyngeal squamous cell carcinoma	[122]
	/	miR-429	in vitro	cervical cancer	[123]
	/	miR-429	in vitro	renal cell carcinoma	[124]
	TβR2/Smad2	miR-200c	in situ, in vitro	endometrioid endometrial carcinoma	[120]
	E2F3/ZEB1	miR-200b	in vivo, in vitro	lung adenocarcinoma	[125]
H19	GIT2/CYTH3	miR-200b/200c	in vivo	breast cancer	[80]
LINC00667	GIT2/CYTH3	miR-200b/200c	in vivo	breast cancer	[80]
XIST	COL1A1/COL3A1/FN1	miR-200c	in situ, in vitro	leiomyoma	[126]
	ZEB1	miR-429	in situ, in vitro	pancreatic cancer	[127]
ATB	Kindlin-2	miR-200b	in situ, in vitro	esophageal squamous cell carcinoma	[128]
ATB	ZNF-217/TGF-β	miR-200c	in situ, in vitro	breast cancer	[129]
OIP5-AS1	FN1	miR-200b	in situ, in vitro	osteosarcoma	[118]
	FOXD1	miR-429	in situ, in vitro	pancreatic ductal adenocarcinoma	[119]
SOX2OT	/	miR-200c	in vitro	bladder cancer	[130]
TMPO-AS1	TMEFF2	miR-200c	in situ, in vitro	ovarian cancer	[131]
	GOT1	miR-429	in situ, in vitro	hepatocellular carcinoma	[132]
DLEU1	TFAP2A	miR-429	in situ, in vitro	ovarian cancer	[133]

**Table 3:** Members of circRNA–miRNA–mRNA ceRNA for miRNAs in subgroup I and subgroup II.

ceRNA Member	Competitor	Shared miRNA	Model Type	Cancer Type	Ref.
hsa_circ_0008305	TIF1γ	miR-200b	in vivo, in situ, in vitro	non-small cell lung cancer	[144]
	TIF1γ	miR-429	in vivo, in situ, in vitro	non-small cell lung cancer	[144]
hsa_circ_0057481	ZEB1	miR-200c	in vitro	laryngeal cancer	[145]
circ_001783		miR-200c		breast cancer	[146]
circ_GNB1	IGF1R axis	miR-141	in situ, in vitro	breast cancer	[147]
circ_101368	HMGB1/RAGE	miR-200a	in situ	hepatocellular carcinoma	[150]
circ_100338	RHEB	miR-141	in situ	hepatocellular carcinoma	[151,152]
circ_ZEB1.33	CDK6	miR-200a	in situ	hepatocellular carcinoma	[153]
circ_SMG1.72	GSN	miR-141	in situ, in vitro	hepatocellular carcinoma	[154]
circ_CRKL	KLF5	miR-141	in situ, in vitro	prostate cancer	[155]
circ_ZEB1	ZEB1	miR-141	in situ, in vitro	prostate cancer	[156]

## Data Availability

Not applicable.
